# Monitoring of Heart and Breathing Rates Using Dual Cameras on a Smartphone

**DOI:** 10.1371/journal.pone.0151013

**Published:** 2016-03-10

**Authors:** Yunyoung Nam, Youngsun Kong, Bersain Reyes, Natasa Reljin, Ki H. Chon

**Affiliations:** 1 Department of Department of Computer Science and Engineering, Soonchunhyang University, Asan, Republic of Korea; 2 Department of Biomedical Engineering, University of Connecticut, Storrs, Connecticut, United States of America; Huazhong University of Science and Technology, CHINA

## Abstract

Some smartphones have the capability to process video streams from both the front- and rear-facing cameras simultaneously. This paper proposes a new monitoring method for simultaneous estimation of heart and breathing rates using dual cameras of a smartphone. The proposed approach estimates heart rates using a rear-facing camera, while at the same time breathing rates are estimated using a non-contact front-facing camera. For heart rate estimation, a simple application protocol is used to analyze the varying color signals of a fingertip placed in contact with the rear camera. The breathing rate is estimated from non-contact video recordings from both chest and abdominal motions. Reference breathing rates were measured by a respiration belt placed around the chest and abdomen of a subject; reference heart rates (HR) were determined using the standard electrocardiogram. An automated selection of either the chest or abdominal video signal was determined by choosing the signal with a greater autocorrelation value. The breathing rate was then determined by selecting the dominant peak in the power spectrum. To evaluate the performance of the proposed methods, data were collected from 11 healthy subjects. The breathing ranges spanned both low and high frequencies (6–60 breaths/min), and the results show that the average median errors from the reflectance imaging on the chest and the abdominal walls based on choosing the maximum spectral peak were 1.43% and 1.62%, respectively. Similarly, HR estimates were also found to be accurate.

## Introduction

Breathing rate (BR) is one of the key vital sign indicators, and is often used to deduce cardiopulmonary health status of a subject. For example, a breathing rate higher than 27 breaths per minute was the most important predictor of cardiac arrest in hospital wards [1]. In unstable patients, relative changes in breathing rate were much greater than changes in heart rate (HR) or systolic blood pressure, and thus the breathing rate was likely to be a better means of discriminating between stable patients and patients at risk [2]. However, in most cases having information on both HR and BR would lead to better diagnosis. Hence, the purpose of this study was to obtain simultaneous measurements of HR and BR from dual cameras of a smartphone.

In the past decade, numerous studies have been proposed in the literature [3–7] for respiratory rate estimation. The most common method for measuring BR is to manually count either the chest wall movements or breath sounds via auscultation with a stethoscope. Previous studies have shown that these manual methods tend to be unreliable in acute care settings, and are limited by their intermittent measurements [8]. For automated approaches to BR assessment, sensors that measure airflow are often used in clinical settings.

Airflow is usually measured using spirometry devices with common sensors, which include the pneumotachograph, nasal cannulae connected to a pressure transducer, a heated thermistor, or anemometry. Airflow can also be measured by detecting either the chest or abdominal movements using respiratory inductance plethysmography (RIP), strain gauges, or magnetometers [3]. Although spirometry devices provide accurate estimates of BR, breathing through a face mask or mouthpiece connected to a pneumotachograph is uncomfortable and adds to the airway resistance. In addition, traditional spirometry devices are inconvenient for mobile applications. There is a need to develop a simple, cost-effective and portable device for estimating BR.

Photoplethysmography (PPG) has also been widely used to estimate respiratory rate due to its simplicity and non-invasive measurement capability [6,9,10] The PPG signal contains components that are synchronous with respiratory and cardiac rhythms. In [6,9–11] respiration is known to modulate the frequency and amplitude of PPG signals. The occurrence of temporal variations in frequency and amplitude is characteristic of the respiration produced by most animals, including humans. Thus, respiratory rates in the normal breathing range can be accurately obtained by computing spectral analysis of the amplitude modulation (AM) and frequency modulation (FM) time series. Recently, several methods for deriving respiratory information from PPG signals using smartphones [7,12,13] have been proposed based on exploiting the characteristics of the respiration-induced modulations in pulse rate, amplitude and width [14]. However, methods based on these modulations have difficulty in detecting high respiratory rates due to several factors, including: 1) the even greater time-varying (TV) nature of these modulations with high respiratory rates; 2) both AM and FM become more subtle and intermittent at high breathing rates, and thus, the highest possible time and frequency resolutions are needed to detect them; and 3) the presence of motion and noise artifacts due to greater body movements can mask AM and FM [4,15,16].

The current gold standard for respiratory rate detection is via the capnograph because it can accurately track the exhaled respiratory gas which has a concentration of carbon dioxide (CO2) 100-fold greater than that of air. However, a capnograph requires calibration, periodic maintenance, and the device is not easily portable. In addition, due to their high cost, capnographs are largely confined to areas of high medical technology use such as operating rooms and intensive care units. Thus, an alternative approach which is less expansive, less cumbersome and easier to use but without compromising the accuracy of respiratory rate and heart rates is desired.

One approach for easy-to-access, affordable and on-demand monitoring of BR and HR is to use smartphones. We have recently shown that accurate estimates of resting BR and HR can be obtained directly from a finger’s pulsatile flows, which are captured using the smartphone’s built-in camera [7]. However, the accuracy of BR estimation degrades at breathing rates higher than 30 breaths/min with this approach. To mitigate this limitation, we propose to use the front camera of a smartphone, hence, a non-contact approach to estimate BRs over a wide dynamic range. Motion analysis using an optical device allows accurate measurement of the kinematics of the chest and abdominal wall in different positions [17,18]. In the past decade, the breathing movement measuring instrument [5,19–22], which consists of six laser distance sensors, has been developed to measure changes in breathing movements of the chest and abdomen. However, it is limited to measuring the anteroposterior diameters of breathing movements, not to mention too complicated to use for general purposes. Although several previous studies have assessed the three-dimensional motions of the chest and abdomen during respiration using infrared cameras [17,18] and an electromagnetic device [23], there is no reported literature on the optical reflectance motion analysis of the observational points on the chest and abdomen using the built-in camera of smartphones. Recently, the vital signs camera application from Philips [24] obtained color changes of a face and shoulder and measured heart rate and breathing rate using only the front camera of a smartphone or a tablet. On the other hand, our approach obtains movements of chest and abdomen as well as fingertip PPG using a front camera and a rear camera concurrently.

## Methods

### Ethics Statement

This study was approved by the institutional review board (IRB) of the Worcester Polytechnic Institute and all participants provided full written consent.

### Data acquisition

**Fig 1:**
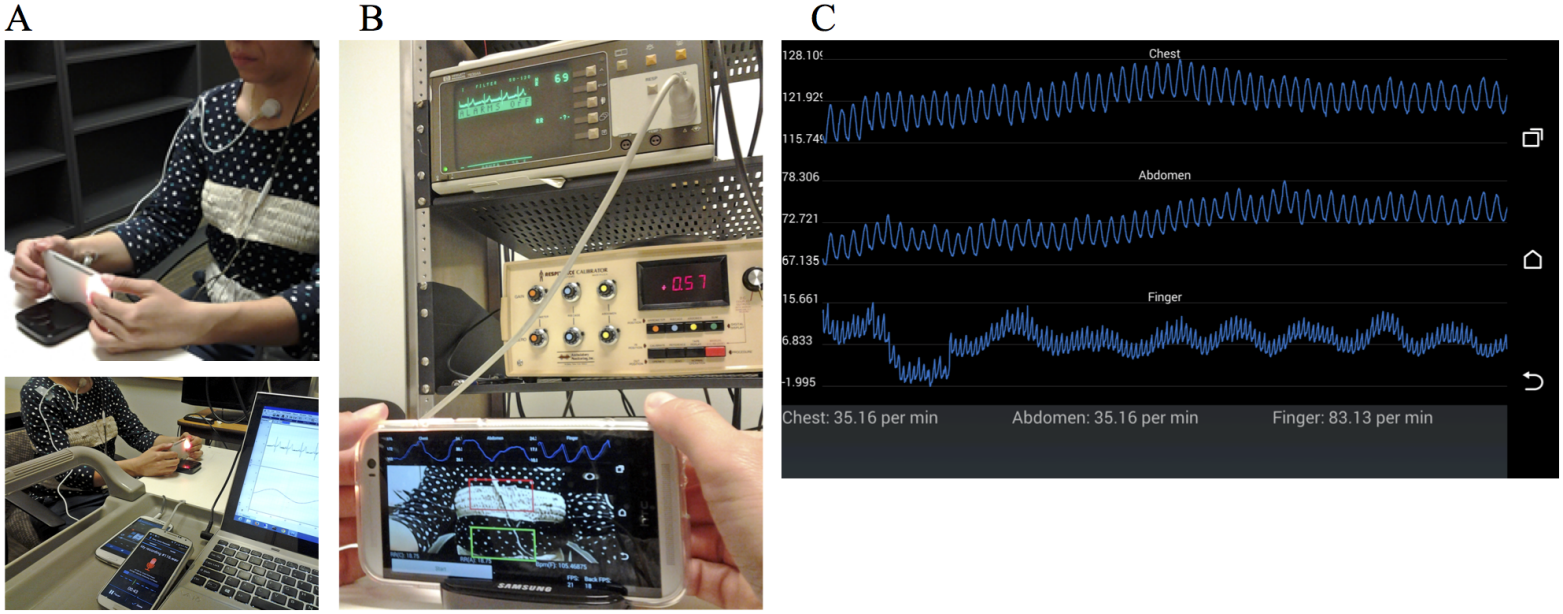
Experimental setup and screenshots of a prototype smartphone application. (A) Experimental setup of smartphone camera positions and subject position. (B) A smartphone prototype application software for estimation of breathing and heart rates. (C) A screenshot of the smartphone application.

Data were collected while healthy volunteers were seated in the upright position. As shown in Fig 1, changes of chest and abdominal movements, as well as a finger’s pulsatile flows, were recorded by the built-in cameras of an HTC One M8 smartphone (HTC Corporation, New Taipei City, Taiwan). For measuring chest and abdominal movements, the experiments were conducted using the front camera. We stabilized the phone on a table to ensure that the camera was not affected by movement during the experiments. The rear camera of the smartphone was used to estimate the heart rate.

To determine reference breathing rates, impedance-based chest belt sensors were wrapped over each subject’s chest and abdomen using the Respitrace system (Ambulatory Monitoring, Inc., Ardsley, NY, USA). As reference for heart rate estimation, electrocardiogram (ECG) recordings were collected with an HP 78354A acquisition system (Hewlett-Packard, Palo Alto, CA, USA) using a standard five-lead electrode configuration. These data were obtained using the LabChart software (ADInstruments Inc., Dunedin, New Zealand) at a sampling rate of 1,000 Hz, while video data were collected directly in the smartphone with digitized sampling rates varying between 20 to 25 Hz (due to inherently variable sampling rates). The maximum sampling rate for the HTC One M8 was 30 frames/sec but can be lower (~25 Hz) due to internal processing load. Due to frame variability, we interpolated the pulsatile signal to 20 Hz and 25 Hz for measuring BR and HR using a cubic spline algorithm, respectively.

The estimated BR and HR were then compared to their corresponding reference signals to test the video cameras’ reliability and accuracy. Specifically, the average magnitude of intensity of inspiration and expiration movements for the chest and the abdomen in the upright position were used to derive estimation of BR.

Data were collected from *N* = 11 healthy non-smokers (2 females and 9 males) between 20 and 40 years old, who were dressed in striped, spotted, or plain clothes. All subjects were instructed to breathe at a metronome rate according to a timed beeping sound programmed at a chosen frequency. The metronome breathing rate was done to test wide dynamics range of breathing rates. Each subject was asked to inhale with each beep sound followed by exhalation before the next beep sound occurred. The data were collected for breathing frequencies ranging from 0.1 to 0.5 Hz at increments of 0.1 Hz, 1 Hz, and spontaneous breathing. Prior to data collection, all subjects were acclimated to different metronome breathing rates. Two minutes of ECG, chest and abdominal movements from the front camera, and finger pulse signals from the rear camera data, were collected for each breathing maneuver, for each subject.

### Preprocessing

**Fig 2:**
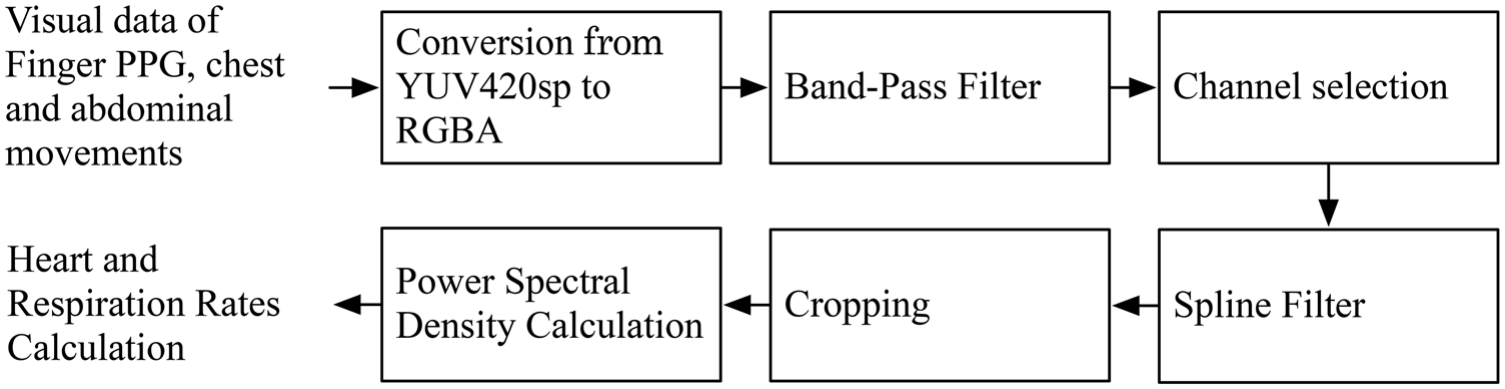
Flowchart of the proposed method for heart and breathing rate estimation using both the front- and rear-facing cameras of a smartphone.

Fig 2 shows the overall flowchart of the proposed method for heart and breathing rates estimation. The first step of the preprocessing of the digitized video signal converts the signal from YUV420sp to RGBA for extracting each channel using the OpenCV library [25]. The second step of the preprocessing involves bandpass filtering (f_low_ = 0.08 Hz and f_high_ = 2.1 Hz for measuring BR, f_low_ = 0.667 Hz and f_high_ = 3.833 Hz for measuring HR). These bandpass filtering parameters were set to account for both normal and abnormal ranges of breathing (0.1–1.0 Hz for breathing and 40–230 bpm for heart rates). The starting and ending 10 seconds of the recordings were discarded. In general, a smartphone provided a sampling rate close to 20–25 frames per second. However, when the video sampling rate was lower than 25 Hz, a cubic spline algorithm was used to interpolate the signal to 20 Hz and 25 Hz for measuring BR and HR, respectively.

### Data Analysis

To extract the experimental data, we set the window size to 120 samples with 50% overlapping segments between consecutive windows at a 20 Hz sampling frequency. Both chest and abdominal reflectance imaging signals consisted of 2,000 samples (2 minutes of data). The BR was determined by using the average power of the optical reflectance imaging signal of either the chest or abdominal area movements.

We used bandpass filtered chest and abdominal reflectance imaging signals to determine which of the two data modalities will provide more accurate estimation of breathing rates by calculating the absolute value of the mean autocorrelation of the chest and abdominal video signals. The criterion was to select the video signal with higher absolute value of the mean autocorrelation since this is a measure of higher signal quality. This allowed us to use an automated procedure for determining which of the two video signals to choose for estimating the breathing rates. Once the video signal was chosen from either the chest or abdomen, the true respiration rate was determined by computing the Welch periodogram of the chosen video signal, and finding the frequency with the maximum amplitude.

To extract optical reflectance imaging signals from chest and abdominal wall movements, two different regions of interest (ROIs) of 49 × 90 pixels were selected out of an area of 320 × 240 pixels focused on the corresponding thoracic area. On the other hand, to obtain the pulse signal derived from a finger placed on the rear camera lens, the vertical left half region closest to the flash of 176 × 72 pixels was selected in a resolution of 176 × 144 pixels for every frame [7].

The intensity value, *I*, of each pixel in the resized image was obtained by averaging the values of red, green, and blue (RGB) channels in the ROI of a single image, as follows:

$$I_{b}\text{(}x,y,t\text{)}=\frac{1}{D}(\sum i_{b}(x,y,t)),$$ (1a)

$$I_{g}\text{(}x,y,t\text{)}=\frac{1}{D}(\sum i_{g}(x,y,t)),$$ (1b)

$$I_{r}\text{(}x,y,t\text{)}=\frac{1}{D}(\sum i_{r}(x,y,t)),$$ (1c)

where *r*, *g*, and *b* are red, green, and blue color band, respectively; *x* and *y* are the coordinates of the image; *t* is time; and *D* is the ROI. For measuring breathing rate, the band with the highest standard deviation among the three color bands (red, green and blue) is selected, while the green channel is selected to measure heart rate [26].

## Results

**Fig 3:**
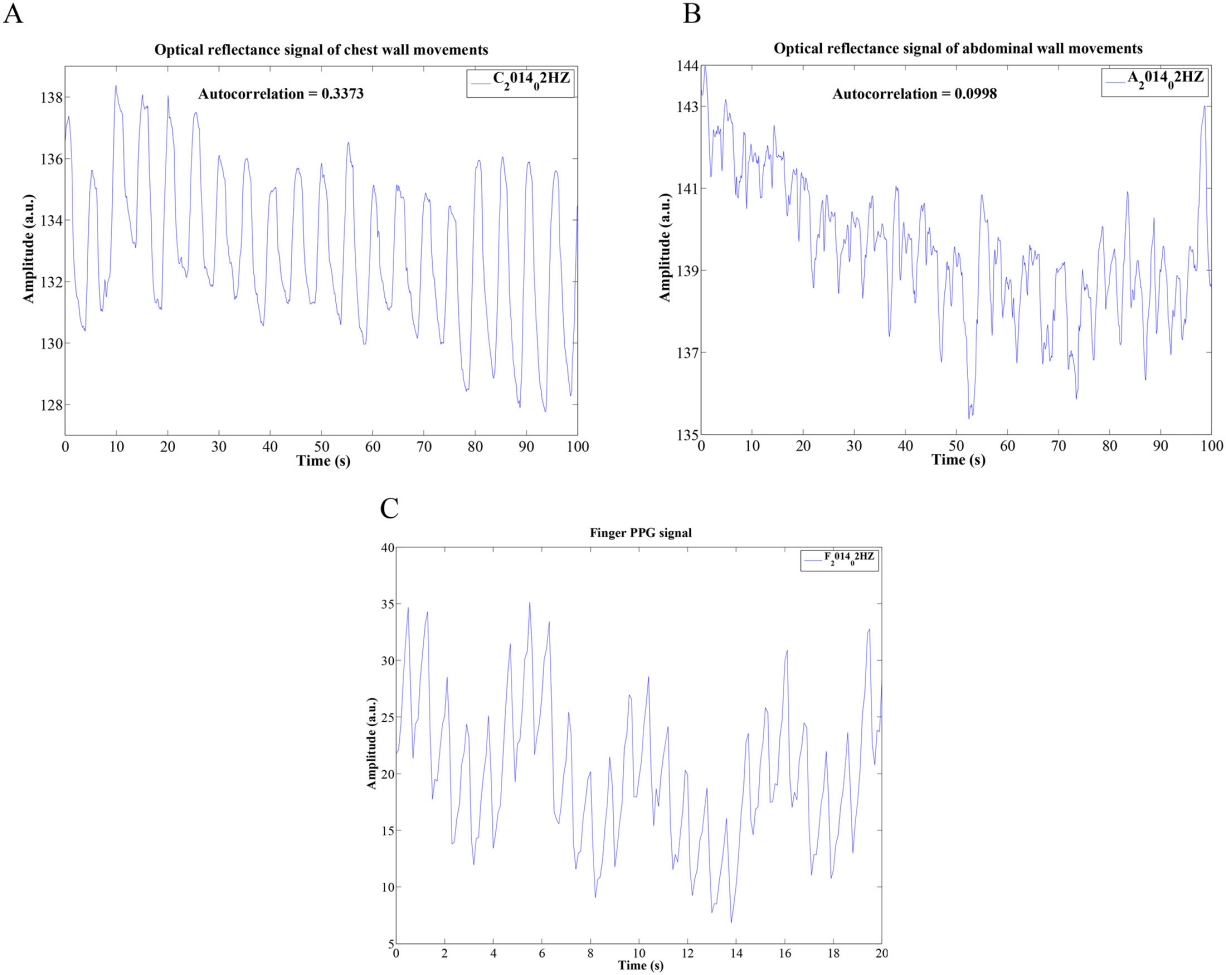
Samples of the recorded chest, abdominal breathing, and finger-PPG signals. (A) optical reflectance imaging signals of chest wall movements. (B) optical reflectance imaging signals of abdominal wall movements. (C) recorded signals of finger-PPG.

Fig 3a and 3b show typical 100-second recordings of the chest and abdomen, respectively, made by the front camera. Fig 3c shows a simultaneous 20-second recording of the finger pulse signal taken by the rear camera of the smartphone. For this particular subject, the optical reflectance imaging signals of chest movements had stronger pulse amplitude and were less affected by noise than were the abdominal wall movements; for some other subjects this was reversed. Indeed chest movements were stronger as reflected by the higher absolute value of the mean autocorrelation value for the chest than for the abdominal video signal, as shown in Fig 3a and 3b. The finger pulse signal shown in Fig 3c is devoid of any motion or noise artifacts.

### Heart Rate Estimation

**Fig 4:**
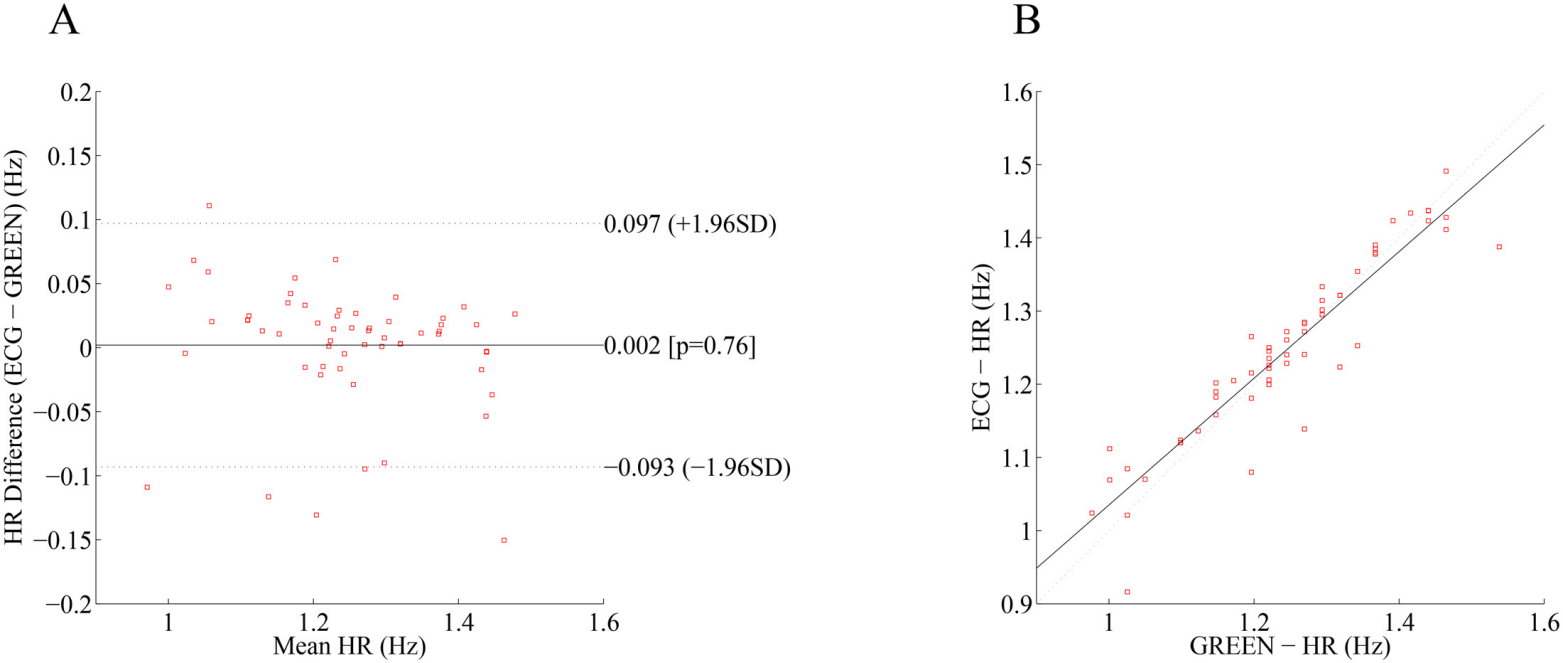
Bland-Altman and correlation plots. (A) An example Bland-Altman plot with a mean difference of 0.002 that shows the limit of agreement of 95% (dashed lines are mean differences ± the limit of agreement) between the continuous HR of a smartphone and its corresponding ECG signal. (B) Example of a correlation plot of the continuous HR monitored from a smartphone (x-axis) and ECG (y-axis) with the regression line (solid line) and a Pearson correlation coefficient of 1 (dashed line).

The Bland-Altman plot is shown in Fig 4(a) for HR data from the HTC One and the reference ECG, and a correlation plot between the HR obtained from the HTC One (green color) camera and the reference ECG HR is shown in Fig 4(b). As shown in the Bland-Altman and correlation plots, both show good agreement in HR between the green color band of the HTC camera and ECG measurements.

### Breathing Rate Estimation

**Fig 5:**
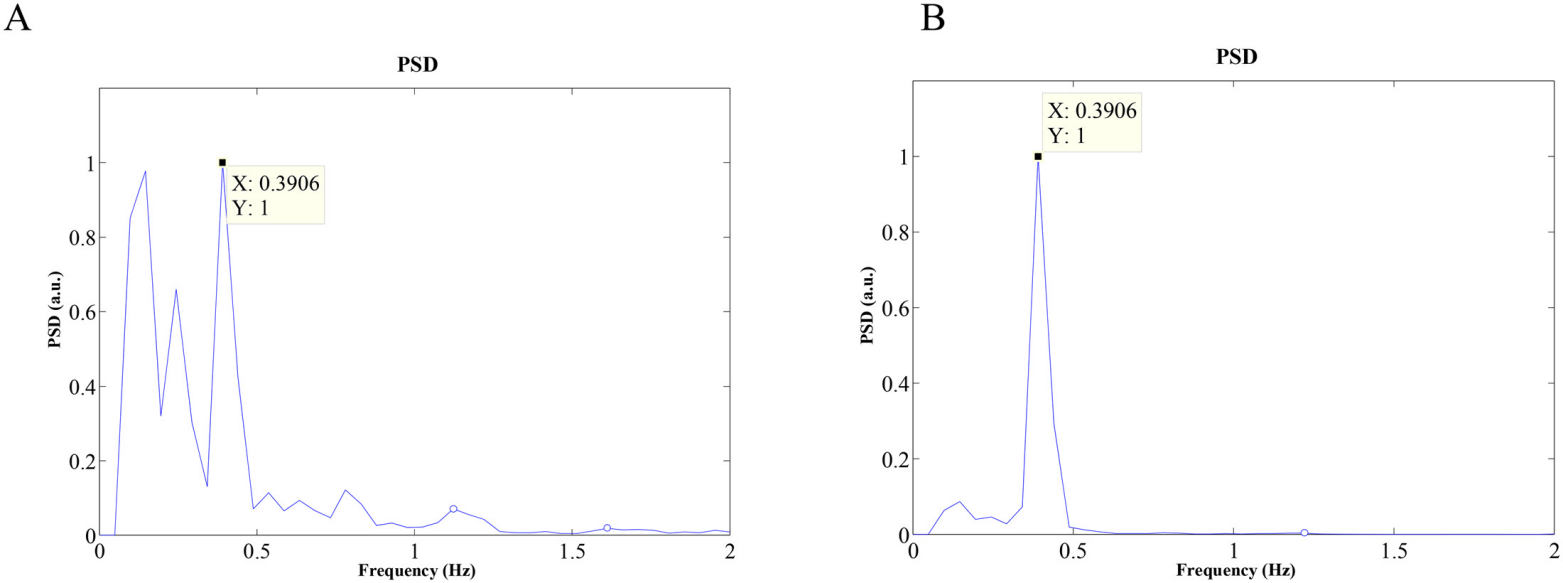
Welch periodogram (normal breathing rate = 0.4 Hz) of: (A) abdomen video signal; (B) chest video signal.

Fig 5a and 5b show Welch periodograms of the abdomen and chest video signals, respectively, for one subject. Since the video signal of the chest has better signal quality than that of the abdomen, there is unequivocally one large peak at the true breathing rate of 0.4 Hz in Fig 5b, while in Fig 5a there are two large peaks at 0.1 Hz and 0.4 Hz. Note that in other subjects, the abdomen has better signal quality than the chest. Hence, in order to determine whether to use the chest or abdomen video signal for estimation of BR, we estimate the absolute value of the mean autocorrelation for both signals and select the signal that has the higher autocorrelation value. Table 1 summarizes the absolute value of the mean autocorrelation values of the chest and abdomen video signals for all subjects. In most subjects, good estimates of breathing rates are found using either the chest or abdomen video signal. However, for subjects 2 and 11 (highlighted in bold underlined font), choosing the higher absolute value of the mean autocorrelation value of the respective video signals results in more accurate breathing rate estimation. Thus, for all subjects, the decision of which of the two video signals to use for accurate breathing rate estimation can be automated by choosing the video signal with higher autocorrelation value.

**Table 1:** Accuracy as determined by median errors from the breathing rate results obtained from chest and abdomen movement signals based on Welch periodogram of the video intensity signal. The absolute value of the mean autocorrelation values of the abdomen and chest video signals are also provided for all subjects.

Breaths/min	Area	Subject 1		Subject 2		Subject 3		Subject 4		Subject 5		Subject 6	
		Welch	Corr value	Welch	Corr value	Welch	Corr value	Welch	Corr value	Welch	Corr value	Welch	Corr value
6 (0.1 Hz)	Abdomen	0.1	0.27	0.1	0.32	0.1	0.15	0.1	0.35	0.1	0.3	0.1	0.16
	Chest	0.1	0.28	0.1	0.33	0.1	0.33	0.1	0.29	0.1	0.32	0.1	0.32
12 (0.2 Hz)	Abdomen	0.2	0.2	0.2	0.36	0.2	0.23	0.2	0.35	0.2	0.36	0.2	0.1
	Chest	0.2	0.34	0.2	0.33	0.2	0.35	0.2	0.29	0.2	0.35	0.2	0.34
18 (0.3 Hz)	Abdomen	0.29	0.23	0.29	0.31	0.29	0.29	0.29	0.33	0.29	0.35	0.29	0.18
	Chest	0.29	0.26	0.29	0.27	0.29	0.33	0.29	0.21	0.29	0.35	0.29	0.33
24 (0.4 Hz)	Abdomen	0.39	0.15	0.39	0.33	0.39	0.3	0.39	0.28	0.39	0.36	0.39	0.34
	Chest	0.39	0.31	0.39	0.31	0.39	0.33	0.39	0.09	0.39	0.35	0.39	0.31
30 (0.5 Hz)	Abdomen	0.49	0.11	**0.15**	**0.07**	0.49	0.32	0.49	0.15	0.49	0.34	0.49	0.15
	Chest	0.49	0.33	**0.49**	**0.26**	0.49	0.32	0.49	0.07	0.49	0.34	0.49	0.13
60 (1 Hz)	Abdomen	0.98	0.19	1.03	0.11	0.98	0.28	0.98	0.27	0.98	0.34	0.98	0.12
	Chest	0.98	0.32	0.98	0.31	0.98	0.3	0.98	0.17	0.98	0.22	0.98	0.23
Breaths/min	Area	Subject 7		Subject 8		Subject 9		Subject 10		Subject 11			
		Welch	Corr value	Welch	Corr value	Welch	Corr value	Welch	Corr value	Welch	Corr value		
6 (0.1 Hz)	Abdomen	0.1	0.34	0.1	0.28	0.1	0.35	0.1	0.22	0.1	0.31		
	Chest	0.1	0.27	0.1	0.16	0.1	0.33	0.1	0.34	0.1	0.31		
12 (0.2 Hz)	Abdomen	0.2	0.35	0.2	0.23	0.2	0.36	0.2	0.36	0.2	0.18		
	Chest	0.2	0.21	0.2	0.26	0.2	0.35	0.2	0.35	0.2	0.22		
18 (0.3 Hz)	Abdomen	0.29	0.34	0.29	0.26	0.29	0.34	0.29	0.19	0.29	0.35		
	Chest	0.29	0.35	0.29	0.19	0.29	0.16	0.29	0.29	0.29	0.31		
24 (0.4 Hz)	Abdomen	0.39	0.35	0.39	0.29	0.39	0.31	0.39	0.19	**0.39**	**0.25**		
	Chest	0.39	0.34	0.39	0.15	0.39	0.16	0.39	0.3	**0.1**	**0.09**		
30 (0.5 Hz)	Abdomen	0.49	0.34	0.49	0.1	0.49	0.32	0.49	0.28	0.49	0.23		
	Chest	0.49	0.23	0.49	0.09	0.49	0.32	0.49	0.31	0.49	0.18		
60 (1 Hz)	Abdomen	0.98	0.27	0.98	0.16	1.03	0.24	0.98	0.28	0.98	0.07		
	Chest	1.03	0.28	0.98	0.2	0.98	0.19	0.98	0.29	0.1	0.06		

To characterize estimation errors of the breathing rates for all subjects using the chest and abdomen signals, and the selected one based on the higher autocorrelation value, we calculated the error ε for each breathing frequency as follows:

$$\varepsilon =\frac{\text{mean(B}-{\text{B}}_{\text{est}}{\text{)}}^{\text{2}}}{{\text{mean(B)}}^{\text{2}}}\times \text{100},$$ (2)

where *B* and *B*_*est*_ represent reference and estimated breathing rate, respectively. The values of error were averaged among all subjects for each breathing maneuver.

**Fig 6:**
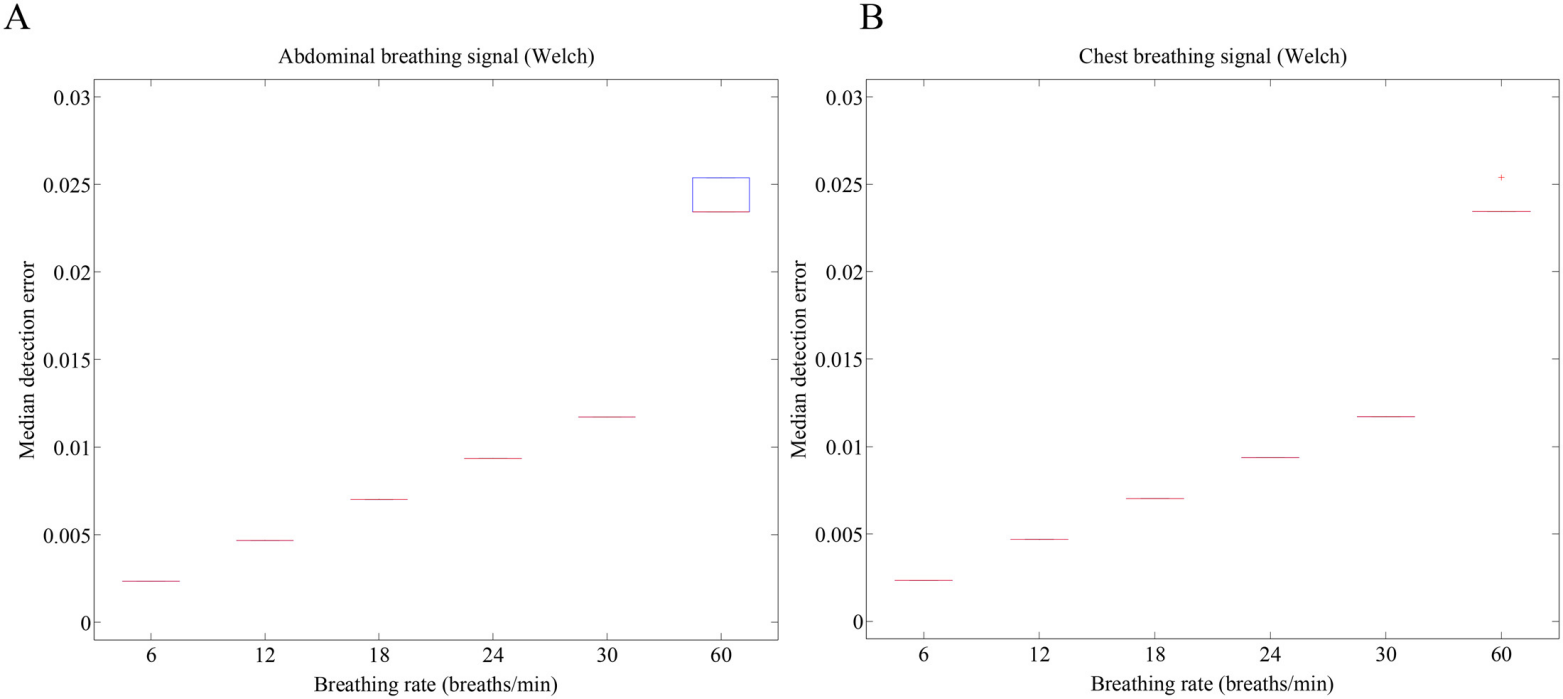
Median and IQR errors measured from the breathing rate results calculated by the maximum peak in PSDs using the Welch periodogram method. (A) Abdominal breathing signal using the Welch periodogram method, (B) Chest breathing signal using the Welch periodogram method.

Fig 6 shows the median and interquartile range (IQR) errors measured from the breathing rate results calculated by the maximum peak in Welch periodogram for both chest and abdomen video signals. The median and IQR errors were obtained from each true and derived respiration rate as defined in Eq 2. The lower boundary of the box closest to zero indicates the 25th percentile, a line within the box marks the median, and the upper boundary of the box farthest from zero indicates the 75th percentile. Whiskers (error bars) above and below the box indicate the 90th and 10th percentiles. Therefore, the area of the blue box is an indication of the spread, i.e., the variation in median error (or IQR) across the population. Since the percentage errors were found to be non-normally distributed, we report the median and IQR values. These figures indicate how well the algorithm performs across the entire population. Red crosses represent the outliers. From the figure, ε was found to be low at all breathing rates. These plots show that the average breathing rate estimation error between the signals of reflectance from imaging of the chest and the abdominal walls is negligible for all breathing rates including a breathing rate as high as 60 breaths/min.

Table 2 summarizes the median and IQR errors measured from the breathing rate results obtained from reflectance imaging on the chest and the abdominal walls, and the video signal selected based on the higher autocorrelation value, for breathing ranges 0.2–0.5 Hz and 1 Hz. From the table, we observe that the average of median errors measured from the breathing rate results obtained from reflectance imaging on the chest and the abdominal walls based on the Welch method using the maximum peak were 1.43% and 1.62%, respectively. When the Welch periodogram was applied to the signal selected by the higher autocorrelation value, the breathing rate measured from reflectance imaging on the abdominal wall provided the lowest median error at all breathing rates. The corresponding average of median errors measured for the breathing rate results obtained from the selected signals of the front camera was reduced to 0.7%. As presented numerically in the table, we observed that the Welch periodogram method combined with signal selection via the absolute value of the mean autocorrelation provides the lowest median error at all breathing rates.

**Table 2:** Accuracy as determined by median errors and IQR measured from the breathing rate results obtained from chest and abdomen movement signals (0.1–0.5 Hz, 1 Hz).

Breathing rate (breathing frequency)	Median/IQR	Welch periodogram method using the maximum peak (%)		Welch periodogram using Corr value (%)
		Abdomen	Chest	
6 bpm (0.1 Hz)	Median	0.23±0.00	0.23±0.00	0.23±0.00
	IQR	0.00±0.00	0.00±0.00	0.00±0.00
12 bpm (0.2 Hz)	Median	0.47±0.00	0.47±0.00	0.47±0.00
	IQR	0.00±0.00	0.00±0.00	0.00±0.00
18 bpm (0.3 Hz)	Median	0.70±0.00	0.70±0.00	0.70±0.00
	IQR	0.00±0.00	0.00±0.00	0.00±0.00
24 bpm (0.4 Hz)	Median	0.94±0.00	3.65±7.67	0.94±0.00
	IQR	0.00±0.00	0.00±0.00	0.00±0.00
30 bpm (0.5 Hz)	Median	4.97±10.74	1.17±0.00	1.17±0.00
	IQR	0.00±0.00	0.00±0.00	0.00±0.00
60 bpm (1 Hz)	Median	2.41±0.09	2.37±0.06	2.37±0.00
	IQR	0.20±0.09	0.00±0.00	0.00±0.00
Average	Median	1.62±1.65	1.43±1.21	0.98±0.68
	IQR	0.03±0.07	0.00±0.00	0.00±0.00

Values presented as mean ± standard deviation.

Table 3 shows the median and IQR errors measured from the breathing rate results obtained from reflectance imaging on the chest and the abdominal walls during spontaneous breathing. True respiration rate was found by computing the PSD of the Respitrace signal and finding the frequency at the maximum amplitude using a respiration belt. From the table, there was no significant difference in the median error between the two methods during spontaneous breathing, and this is why the results when selecting the signal with higher autocorrelation value are not presented.

**Table 3:** Accuracy as determined by median errors and IQR measured from the breathing rate results obtained from chest and abdomen movement signals (spontaneous).

Breathing maneuver	Median/IQR	Welch periodogram method using the maximum peak (%)	
		Chest	Abdomen
Spontaneous	Median	3.80±2.03	3.80±2.03
	IQR	2.44±1.15	2.44±1.15

Values presented as mean ± standard deviation.

## Discussion and Conclusion

In this paper, we presented an approach to simultaneously estimate heart and breathing rates using both the front- and rear-facing cameras of a smartphone. The HR is estimated via a contact approach by placing a finger on top of the rear-facing camera while the BR is determined via non-contact imaging of the chest and abdominal movements using the front-facing video camera.

HR estimation via the imaging of a fingertip showed comparable results to those reported in the literature [26–31]. In particular, we found a non-statistically-significant bias of 0.12 beats-per-minute and 95% limits of agreement of -5.58 to 5.52 beats-per-minute when compared to the reference ECG derived heart rates. It is worth mentioning that emphasis of this study was not on the analysis of the HR estimation but on the BR instead as the former has been the focus of interest of many researchers. The aim was to show that similar to previous studies, good HR estimation results can be obtained even when simultaneously BR is collected using an additional camera sensor on the smartphone.

For BR estimation, several non-contact approaches have been proposed in the literature [32–37]. Poh *et al*. found high correlation of BR and HR estimates extracted via independent component analysis (ICA) of webcam acquired video signals and their corresponding reference values [32]. However, the breathing rates were confined to normal breathing ranges (10–20 breaths/min) [32]. A custom-built system consisting of a monochrome camera and an infrared illumination source was employed by Sun *et al*. to successfully extract BR and HR in exercise conditions by applying an artifact reduction method based on single-channel ICA [33]. Wu *et al*. employed an Eulerian-based method combining spatial and temporal processing that can be used to magnify small motions around the chest related to breathing in video sequences [34]. Zhao *et al*. also estimated BR and HR via dynamic embedding technique and ICA applied to single channel video recordings and enhanced the results by removing false signals [35]. Tarassenko *et al*. employed autoregressive modeling and pole selection to estimate HR and BR from the analysis of RGB video signals of patients in clinical environments [36]. Recently, Shao *et al*. estimated, among other parameters, HR and BR by analyzing skin color changes and body movements of the shoulder, respectively, with the results found to be in agreement with the corresponding reference signals [37]. While all of the aforementioned methods provide good estimates of BR for normal breathing ranges (10–22 breaths/min), it remains to be seen if their accuracy is retained for higher breathing rates. Moreover, these methods have been designed for real-time implementation using personal computers and employ custom-built video recording systems. To our best knowledge, the simultaneous estimation of HR via a contact approach and BR via a non-contact approach directly implemented on a commercially available smartphone has not been done.

The results of our study indicate that for both low and high breathing ranges (0.1–1 Hz), the video camera signal from a smartphone can be effectively used to identify changes in breathing movements of the chest and abdomen compartments. We compared both the chest and abdominal motions acquired with smartphones for BR estimation. In general, we found accurate breathing estimates in both the normal and high breathing ranges. We found that the approach using the maximum peaks based on Welch periodogram provided accurate breathing rate estimates from either chest or abdominal movements, for both low and high breathing rates. However, in some medium to high breathing rates (0.4 and 0.5 Hz), a simple approach using the maximum peak detection did not always provide satisfactory results, especially when subjects wore either loose clothing or breathed shallowly. To this end, we proposed a method to automatically select the signal from either the abdomen or the chest, whichever video signal has a greater absolute value of mean autocorrelation. Our results shown in Tables 1 and 2 indicate that selecting a video signal with a higher absolute value of the mean autocorrelation value automates the choice of which video signal to choose and provides more accurate BR estimates.

**Fig 7:**
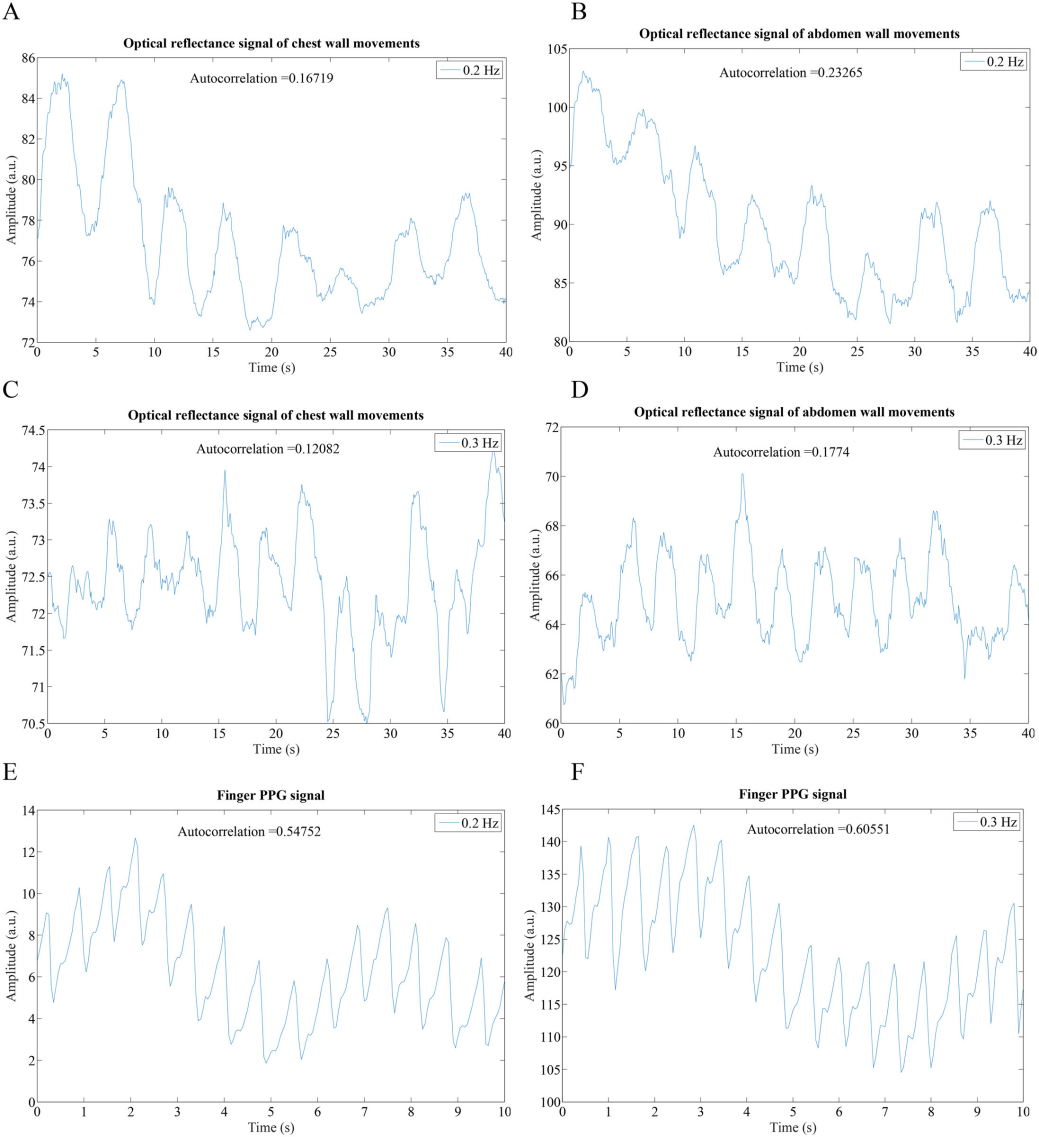
Representative chest and abdominal breathing, and finger-PPG signal recordings with the subjects in standing posture. (A & B) Optical reflectance imaging signals of chest wall movements at 0.2 Hz and 0.3 Hz breathing rates, respectively. (C & D) Optical reflectance imaging signals of abdominal wall movements at 0.2 Hz and 0.3 Hz breathing rates, respectively. (E &D) Finger-PPG at 0.2 Hz and 0.3 Hz breathing rates, respectively.

**Fig 8:**
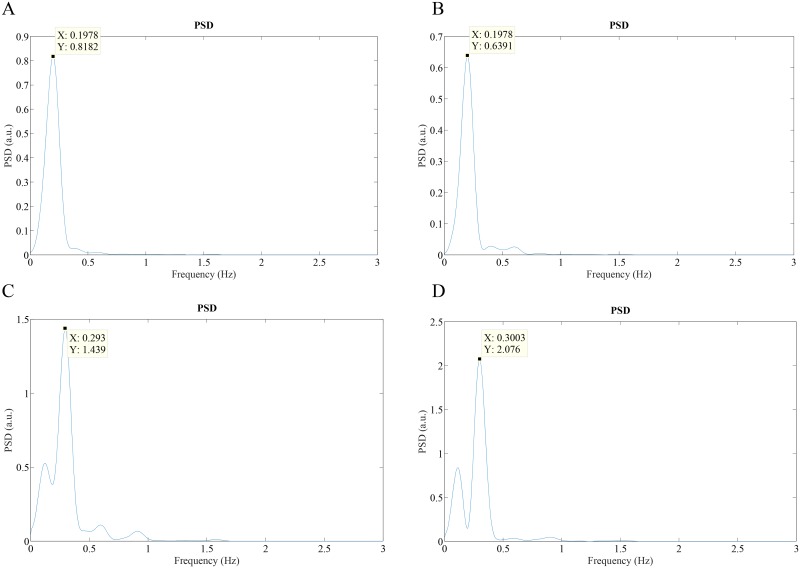
Welch periodogram (BR = 0.2 Hz and 0.3 Hz) of: (A) Chest video signal (BR = 0.2 Hz); (B) Abdomen video signal (BR = 0.2 Hz); (C) Chest video signal (BR = 0.3 Hz); (D) Abdomen video signal (BR = 0.3 Hz).

One limitation of the current study is that we have minimized movement of hands during data recording which may not be always feasible. As shown in Fig 1, the movement of the hands was minimized by requiring subjects to rest the smartphone on a table during data collection. However, we have found that with some training, some subjects are able to collect both video signals without resting their hands on a table. To further demonstrate the feasibility of BR estimation without having the camera fixed in an immobile position, we conducted experiments with subjects in the standing posture. Fig 7a–7d show typical 40-second recordings of the chest and abdomen, respectively, recorded with the front camera. Fig 7e and 7f show a simultaneous 10-second recording of the finger pulse signal taken by the rear camera of the smartphone. Fig 8a–8d show Welch periodograms of the chest and abdomen video signals shown in Fig 7. As shown, BR can be accurately estimated from non-contact video recordings from both chest and abdominal motions while subjects were in standing still posture. However, a motion artifact correction algorithm such as the one recently developed by Wang et al. [38] can be implemented in real time to eliminate motion artifacts. In addition, we have found that variations in colors and patterns in a shirt provide higher fidelity video recordings. For example, we have noticed that when a subject was dressed in plain clothes, relative changes in the chest and abdominal movements from the ROI of the front camera were considerably smaller than when a subject was dressed in striped or non-uniform color clothes.

In conclusion, our proposed approach allows accurate attainment of two vital sign measurements directly from a smartphone by using its dual cameras simultaneously: the heart rate and breathing rate, whereby the estimation of one vital sign by a camera sensor did not have deleterious effect on the results of the second video camera.
